# Supplementary material for: The atypical antipsychotic aripiprazole alters the outcome of disseminated Candida albicans infections
Source: bioRxiv. 2024 Feb 14:2024.02.13.580133. Preprint. [Version 1] doi: 10.1101/2024.02.13.580133 (PMC10888916; doi:10.1101/2024.02.13.580133)
Supplement: Supplement 1 [file NIHPP2024.02.13.580133v1-supplement-1.pdf]

bioRxiv preprint doi: https://doi.org/10.1101/2024.02.13.580133; this version posted February 14, 2024. The copyright holder for this preprint (which was not certified by peer review) is the author/funder, who has granted bioRxiv a license to display the preprint in perpetuity. It is made available under aCC-BY 4.0 International license.

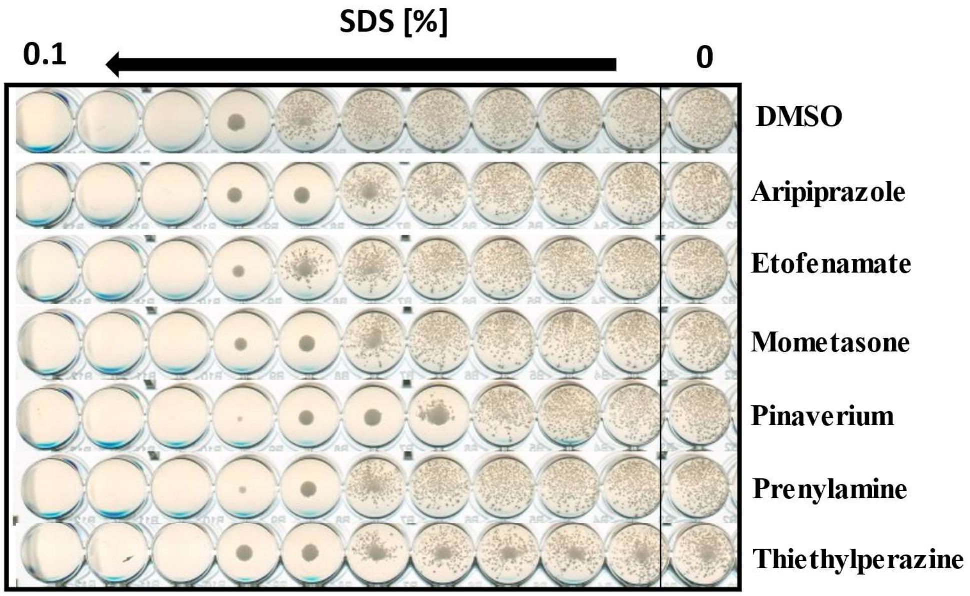

Figure S1. Several antifungal antagonists decrease tolerance to the cell membrane stressor SDS. Stressor susceptibility assays were set conducted with *C. albicans* SC5314 in 96-well plates at 1 x 10^4 cells/ml in RPMI-pH 7 containing increasing 2-fold concentrations of the cell membrane stressor SDS. Cells were grown in the presence of either DMSO or 5 µM of each medication, and then incubated at 35°C. Plates were imaged at 24-hours.

bioRxiv preprint doi: https://doi.org/10.1101/2024.02.13.580133; this version posted February 14, 2024. The copyright holder for this preprint (which was not certified by peer review) is the author/funder, who has granted bioRxiv a license to display the preprint in perpetuity. It is made available under aCC-BY 4.0 International license.

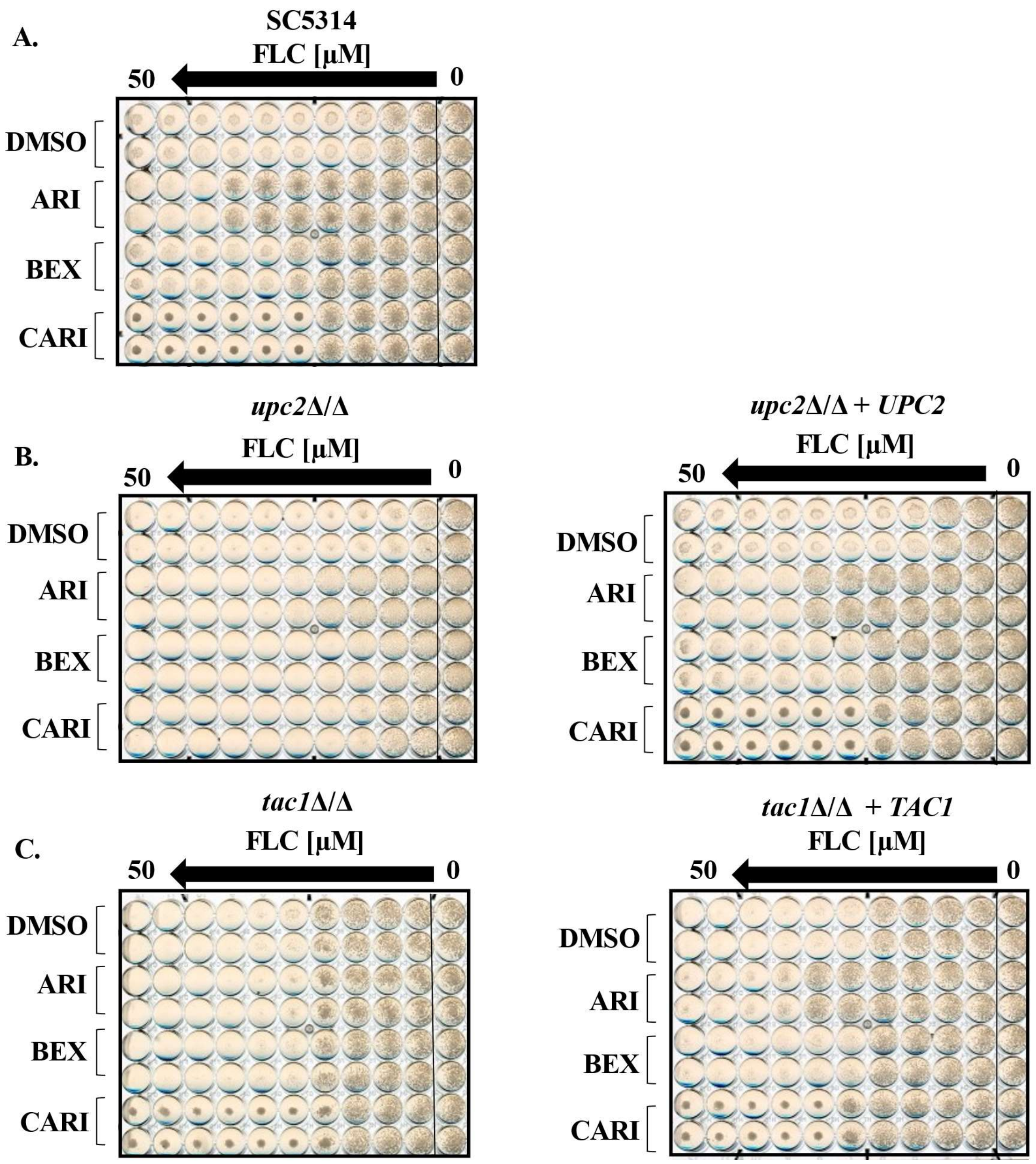

Figure S2. Aripiprazole analogs act through distinct mechanisms. A. Fluconazole (FLC) susceptibility assays were performed with *C. albicans* strain SC5314 using the CLSI method. Medium was supplemented with either 5 µM aripiprazole (ARI), bexpiprazole (BEX), cariprazine (CARI), or 0.5% DMSO (vehicle control) and imaged after 24-hours of incubation. Plates are representative of assays performed in biological duplicate. **B-C.** Fluconazole susceptibility assays were performed as described above with a *upc2*Δ/Δ strain and *upc2*Δ/Δ + *UPC2* derived strain (**B.**) or *tac1*Δ/Δ and its *tac1*Δ/Δ + *TAC1* derived strain accordingly (**C.**). Plates are representative of assays performed in biological duplicate.

bioRxiv preprint doi: https://doi.org/10.1101/2024.02.13.580133; this version posted February 14, 2024. The copyright holder for this preprint (which was not certified by peer review) is the author/funder, who has granted bioRxiv a license to display the preprint in perpetuity. It is made available under aCC-BY 4.0 International license.

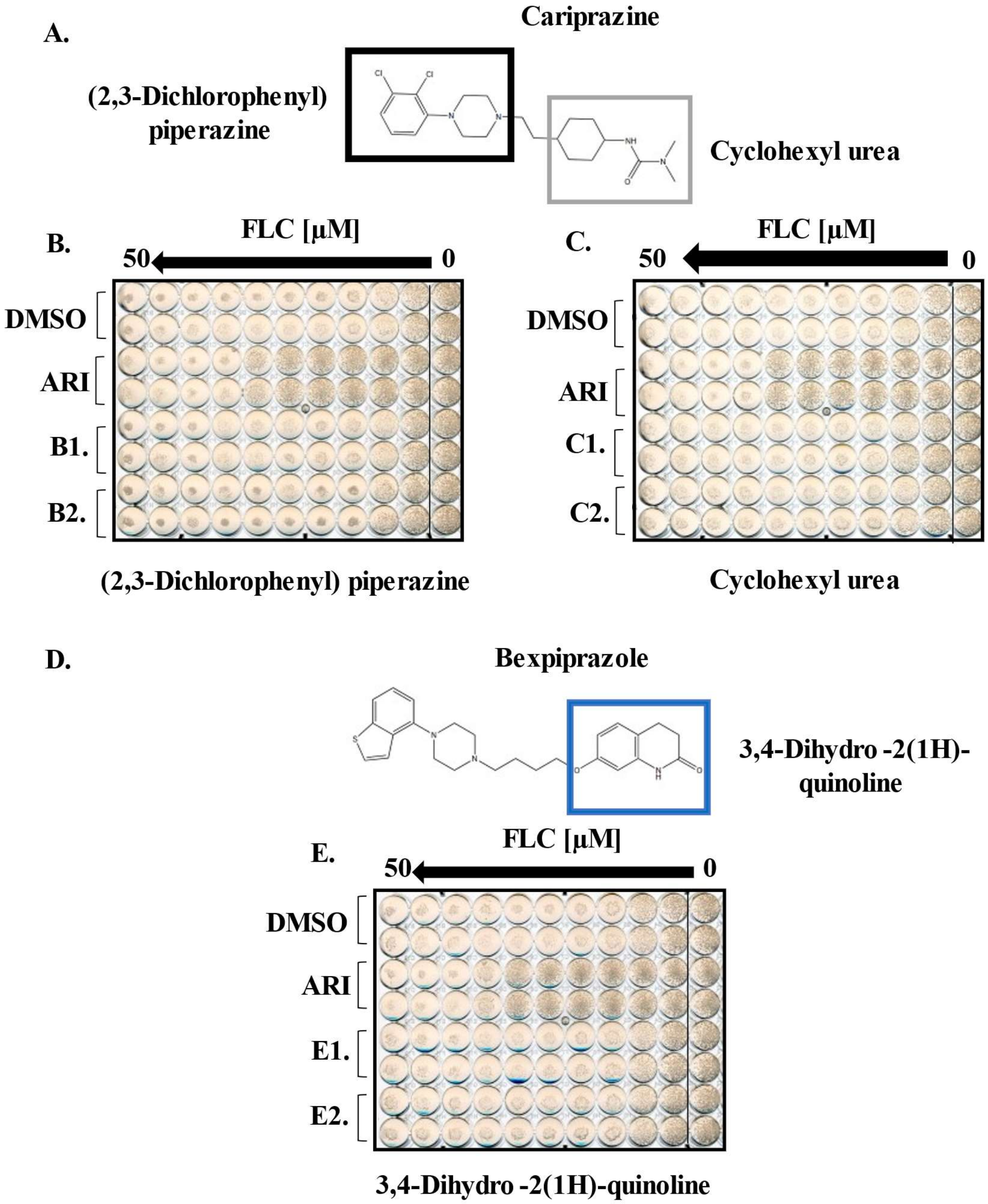

Figure S3. Cariprazine and bexpiprazole sub-structures alone are insufficient to alter fluconazole activity against *Candida albicans*. A. The chemical structure of cariprazine with the two sub-structures highlighted: (2,3-dichlorophenyl)piperazine (indicated by a black square) or cyclohexyl urea (indicated by a gray square). **B.** Fluconazole (FLC) susceptibility assays were performed with *C. albicans* strain SC5314 using CLSI conduct. Medium was supplemented with either 0.5% (DMSO) 5 μM aripiprazole (ARI), 5 μM (**B1.**), or 25 μM (**B2.**) of (2,3-Dichlorophenyl) piperazine and plates imaged after 24-hours of incubation. Plates are representative of assays performed in biological duplicate. **C.** FLC susceptibility assays were performed as described in (**B.**), using the substructure cyclohexyl at two concentrations: 5 μM (**C1.**) or 25 μM (**C2**). **D.** Bexpiprazole containing the sub-structure 3,4-Dihydro-2(1H)-quinoline (indicated by a blue square). **E.** FLC susceptibility assays were performed as described in (**B.**), with using the substructure 3.4-dihydro-2(1H)-quinoline at 5 μM (**E1.**) or 25 μM (**E2.**).

bioRxiv preprint doi: https://doi.org/10.1101/2024.02.13.580133; this version posted February 14, 2024. The copyright holder for this preprint (which was not certified by peer review) is the author/funder, who has granted bioRxiv a license to display the preprint in perpetuity. It is made available under aCC-BY 4.0 International license.

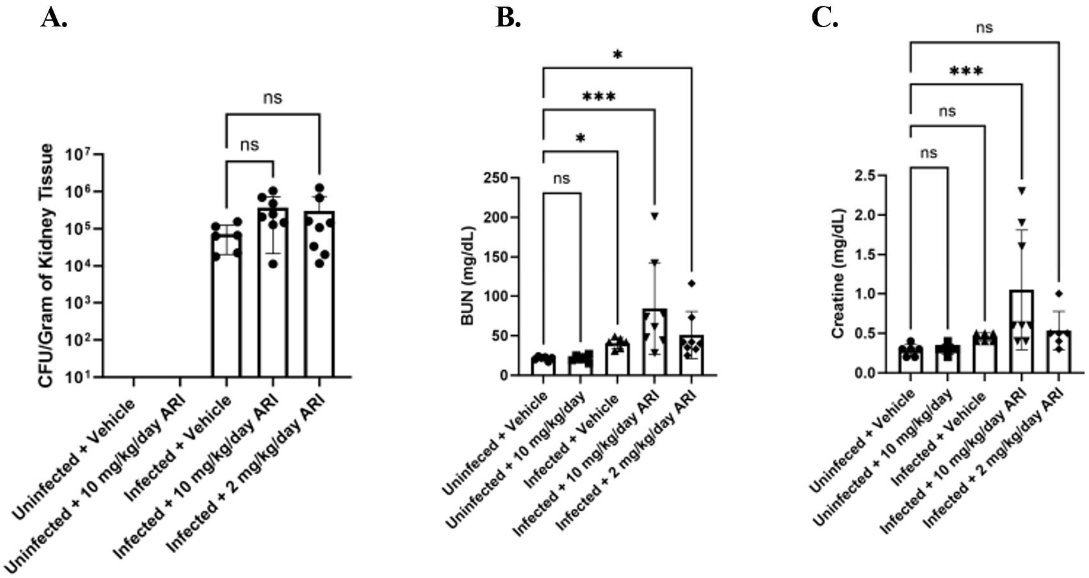

Figure S4. Aripiprazole effects disease progression in C57BL/6 mice. A. Groups of C57BL/6 mice (n = 8) were treated daily with either 10 or 2 mg/kg/day aripiprazole or vehicle starting 3 days prior to infection and then infected with 2.5 x 10^5 of SC5314 or PBS (vehicle control). At 48-hour endpoints, kidneys were excised, and fungal burden quantified as CFU per gram of tissue. Data is depicted as the mean + standard deviation. Statistical significance was calculated using a one-way ANOVA and Kruskal-Wallis post-test. **B-C.** Blood was extracted from the same mice as in (**A.**) serum processed, and blood urea nitrogen (BUN) (**B.**) or creatinine levels (**C.**) were quantified. Groups were compared to uninfected vehicle group. Data is depicted as the mean + standard deviation, and statistical significance was determined using a one-way ANOVA and Kruskal-Wallis post-test (n = 8).
* $P < 0.05$, *** $P < 0.0005$. ns = not significant

bioRxiv preprint doi: https://doi.org/10.1101/2024.02.13.580133; this version posted February 14, 2024. The copyright holder for this preprint (which was not certified by peer review) is the author/funder, who has granted bioRxiv a license to display the preprint in perpetuity. It is made available under aCC-BY 4.0 International license.

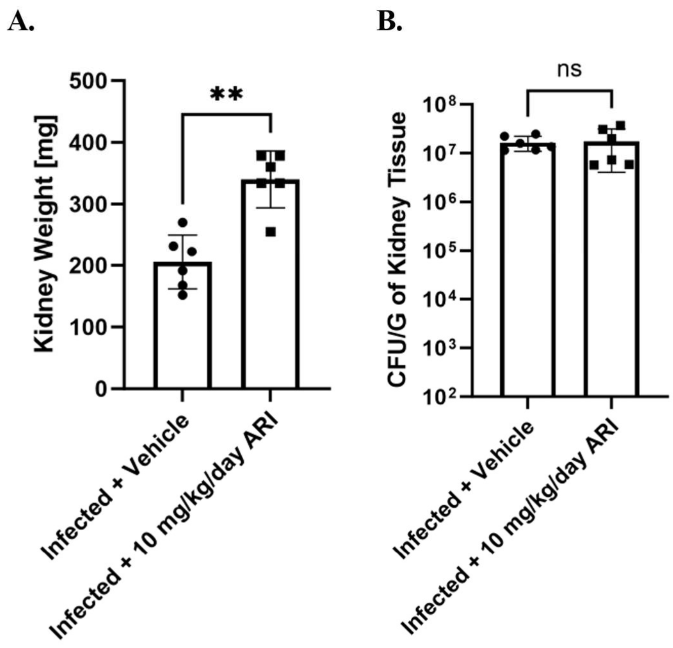

Figure S5: Aripiprazole exacerbates disease in a disseminated candidiasis infection. A. Groups of BALB/c mice (n = 6) were treated daily with 10 mg/kg/day aripiprazole (ARI) or vehicle starting 3 days prior to infection. Mice were then infected with 4 x 10^5 of the CAI4+pKE4-NRG1 yeast-locked strain, and 5 days p.i, mice were euthanized, kidneys were excised. Kidney weight was recorded, and weight was compared between drug and vehicle treated groups. Data is depicted as the mean + standard deviation, and statistical significance calculated using a one-way ANOVA and Mann-Whitney post-test. B. Kidneys from mice in (**A.**) were homogenized, and fungal burden was quantified as CFU per gram of tissue. Data is depicted as the mean + standard deviation, and statistical significance calculated using a one-way ANOVA and Mann-Whitney post-test .

** $P < 0.005$. ns = not significant

bioRxiv preprint doi: https://doi.org/10.1101/2024.02.13.580133; this version posted February 14, 2024. The copyright holder for this preprint (which was not certified by peer review) is the author/funder, who has granted bioRxiv a license to display the preprint in perpetuity. It is made available under aCC-BY 4.0 International license.

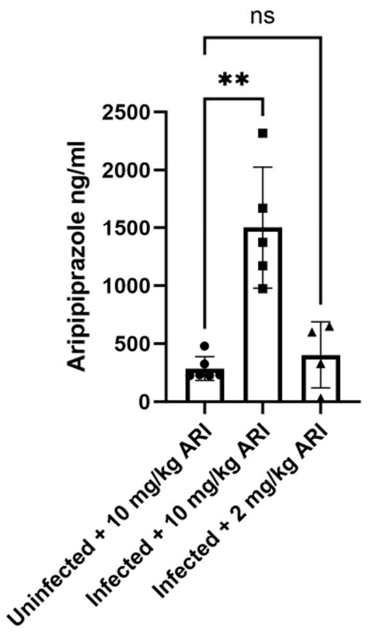

Figure S6: Aripiprazole concentration increases during disseminated candidiasis infection. Groups of BALB/c mice (n = 6) were treated daily with 10 or 2 mg/kg/day aripiprazole (ARI) starting 3 days prior to infection. Mice were then infected with 2.5 x 10^5 of SC5314, and drug treatment continued. At the 48-hour endpoint, blood was collected, plasma extracted, and aripiprazole concentration was quantified using LC-MS. Infected drug treated mice were compared to uninfected treated mice. Data is depicted as the mean + standard deviation, and statistical significance was calculated using a one-way ANOVA and Kruskal-Wallis post-test.
** $P < 0.005$. ns = not significant

bioRxiv preprint doi: https://doi.org/10.1101/2024.02.13.580133; this version posted February 14, 2024. The copyright holder for this preprint (which was not certified by peer review) is the author/funder, who has granted bioRxiv a license to display the preprint in perpetuity. It is made available under aCC-BY 4.0 International license.

		Log_2 of Average Fold Change	
**Gene name**	**Gene description**	**Aripiprazole**	**Etofenamate**
RDN18	18S ribosomal RNA; component of the small (40S) ribosomal subunit	4.983	4.89
ADH3	Putative NAD-dependent (R,R)-butanediol dehydrogenase	4.112	3.3
PDR16	Phosphatidylinositol transfer protein; induction correlates with *CDR1*, *CDR2* overexpression/azole resistance	2.936	1.787
CDR1	Multidrug transporter of ABC superfamily; transports phospholipids in an in-to-out direction	2.419	1.546
SEO1	Protein with similarity to permeases	2.214	-1.672
orf19.4907	Putative protein of unknown function; Hap43p -repressed gene; increased transcription is observed upon fluphenazine treatment; possibly transcriptionally regulated by Tac1p fungal -specific (no human/murine homolog)	2.096	1.305
MRV2	Protein of unknown function; repressed by fluphenazine treatment or in an azole-resistant strain that overexpresses *CDR1* and *CDR2*	-3.073	-1.633
CFL5	Ferric reductase; induced in low iron; ciclopirox olamine, flucytosine induced; amphotericin B, Sfu1 repressed	-3.532	-1.841

Table S1. Overlap of aripiprazole and etofenamate responsive genes. *C. albicans* was grown in RPMI-pH 7 at 35ºC for 6-hours with 5 µM of aripiprazole, etofenamate, or 0.5% DMSO (vehicle). High throughput sequence analysis was performed, and significantly altered (log_2fold > 1 or < -1, adjusted to p < 0.5) gene expression (compared to vehicle controls) determined.

bioRxiv preprint doi: https://doi.org/10.1101/2024.02.13.580133; this version posted February 14, 2024. The copyright holder for this preprint (which was not certified by peer review) is the author/funder, who has granted bioRxiv a license to display the preprint in perpetuity. It is made available under aCC-BY 4.0 International license.

		Log$_2$ of Average Fold Change	
**Gene name**	**Gene description**	**Aripiprazole**	**Mometasone**
orf19.344	Protein of unknown function; upregulated by fluphenazine treatment or in an azole -resistant strain that overexpresses CDR1 and CDR2	2.629	1.588
MET3	ATP sulfurlyase ; sulfate assimilation; repressed by Met, Cys, Sfu1, or in fluconazole -resistant isolate	-1.511	-1.6
orf19.4654	Protein of unknown function	-2.654	-2.839
JEN2	Dicarboxylic acid transporter; regulated by glucose repression	-3.769	-2.644
orf19.4653	Protein like GPI -linked cell -wall proteins	-8.838	-2.667

Table S2. Overlap of aripiprazole and mometasone responsive genes. *C. albicans* was grown in RPMI-pH 7 at 35ºC for 6-hours with 5 µM of aripiprazole, mometasone, or 0.5% DMSO (vehicle). High throughput sequence analysis was performed, and significantly altered ($\log_2$fold > 1 or < -1, adjusted to $p < 0.5$) gene expression (compared to vehicle controls) determined.

		Log$_2$ of Average Fold Change	
**Gene name**	**Gene description**	**Etofenamate**	**Mometasone**
snR57b	C/D box small nucleolar RNA (snoRNA)	-1.244	-1.606

Table S3. Overlap of etofenamate and mometasone responsive genes. *C. albicans* was grown in RPMI-pH 7 at 35ºC for 6-hours with 5 µM of etofenamate, mometasone, or 0.5% DMSO (vehicle). High throughput sequence analysis was performed, and significantly altered ($\log_2$fold > 1 or < -1, adjusted to $p < 0.5$) gene expression (compared to vehicle controls) determined.

bioRxiv preprint doi: https://doi.org/10.1101/2024.02.13.580133; this version posted February 14, 2024. The copyright holder for this preprint (which was not certified by peer review) is the author/funder, who has granted bioRxiv a license to display the preprint in perpetuity. It is made available under aCC-BY 4.0 International license.

Gene name	Gene description	Log_2 of Average Fold Change
orf19.5785	Protein of unknown function; upregulated in a cyr1 or ras1 null mutant; induced by nitric oxide	3.613
RDN25	25S ribosomal RNA; component of the large (60S) ribosomal subunit; encoded in about 55 copies of the rDNA repeat on Chromosome R; in some strains the gene may contain the self-splicing group I intron	3.253
orf19.22.1	Pseudogene; added to Assembly 21 based on comparative genome analysis	3.089
orf19.1782.1	Protein of unknown function	2.978
orf19.6200	Pry family pathogenesis-related protein; oral infection upregulated gene; mutant has reduced capacity to damage oral epithelial cells	2.778
orf19.1430	Protein of unknown function	2.500
IFD6	Aldo-keto reductase; similar to aryl alcohol dehydrogenases; protein increase correlates with MDR1 overexpression (not CDR1 or CDR2) in fluconazole-resistant clinical isolates; farnesol regulated; possibly essential	2.497
orf19.7042	Protein of unknown function; induced by benomyl or in an azole-resistant strain overexpressing MDR1	2.346
OP4	Ala- Leu- and Ser-rich protein; secreted; N-terminal hydrophobic region; possible glycosylation; opaque-specific transcript; repressed by alpha pheromone in opaque MTLa homozygotes; fluconazole-induced	1.888
RPR1	Putative ortholog of S. cerevisiae RNAse P RNA; gene transcribed by RNA Pol III	1.851
CSP37	Hyphal cell wall protein; role in progression of mouse systemic infection; predicted P-loop, divalent cation binding, N-glycosylation sites; expressed in yeast and hyphae; hyphal downregulated	1.827
CPD2	Protein with homology to NADH dehydrogenase; regulated by Sef1p-, Sfu1p-, and Hap43p	1.821
orf19.6501	Protein of unknown function	1.720
orf19.1887	Ortholog(s) have sterol esterase activity, role in sterol metabolic process and integral component of membrane, lipid droplet localization	1.622
TAC1	Zn(2)-Cys(6) transcriptional activator of drug-responsive genes (CDR1 and CDR2); binds DRE element; gene in zinc cluster region near MTL locus	1.602
MNN42	Protein of unknown function; repressed by Rim101; negatively modulates intracellular ATP levels during the development of azole resistance; induced by Ca(2+) in a calcineurin-dependent manner	1.563
orf19.4459	Predicted heme-binding stress-related protein; Tn mutation affects filamentous growth	1.540
orf19.1862	Possible stress protein; increased transcription associated with CDR1 and CDR2 overexpression or fluphenazine treatment; regulated by Sfu1, Nrg1, Tup1; stationary phase enriched protein	1.523
orf19.86	Putative glutathione peroxidase; induced by peroxide, exposure to neutrophils and macrophage blood fractions; repressed during infection of macrophages	1.488
CFL11	Superoxide-generating NADPH oxidase, produces extracellular burst of reactive oxygen species at growing cell tips during hyphal morphogenesis	1.483
YMX6	Putative NADH dehydrogenase; macrophage-downregulated gene; induced by nitric oxide	1.466
LCB4	Putative sphingosine kinase; Tac1p-regulated expression	1.463
HSP12	Heat-shock protein; induced by osmotic/oxidative/cadmium stress, fluphenazine treatment, low iron, CDR1 and CDR2 overexpression, or ssn6 or ssk1 null mutation; overexpression increases resistance to farnesol and azoles	1.460
YWP1	Secreted yeast-cell wall protein; controls exposure of cell wall beta-glucan to host immune system; involved in adhesion and biofilm formation; growth phase	1.434
YOR1	Oligomycin resistance ATP-dependent permease YOR1	1.389
IFU5	Predicted membrane protein involved in cell wall maintenance; estradiol-induced; upregulation associated with CDR1 and CDR2 overexpression or fluphenazine; putative drug-responsive regulatory site; Hap43p-repressed; Tac1p-regulated	1.376
orf19.2726	Putative plasma membrane protein; Plc1-regulated	1.334
PGA17	utative GPI-anchored protein; exogenously expressed protein substrate for Kex2 processing in vitro; repressed by alpha pheromone in SpiderM medium; macrophage-induced; induced in oralpharyngeal candidiasis	1.124
orf19.4668	Protein with a glycoside hydrolase domain; mutants are viable	-1.116
PGA34	Putative GPI-anchored protein;; induced in oralpharyngeal candidasis	-1.159
orf19.5140	Protein of unknown function	-1.329
orf19.2633.1	Protein of unknown function	-1.376
CFL4	C-terminus similar to ferric reductases; induced in low iron; Sfu1-repressed; ciclopirox olamine induced; colony morphology-related gene regulation by Ssn6; Hap43-repressed; Sef1-regulated	-1.450
UME6	Zn(II)2Cys6 transcription factor; has a long 5'-UTR that regulates translational efficiency and controls transition to filamentous growth; stability controlled by Grr1p, Ubr1p, Ptc2p in response to CO2 and O2 levels	-1.604
orf19.3897	Protein of unknown function; decreased transcription is observed upon fluphenazine treatment or in an azole-resistant strain that overexpresses CDR1 and CDR2	-1.668
IHD1	GPI-anchored protein; alkaline, hypha-induced; regulated by Nrg1, Rfg1, Tup1 and Tsa1, Tsa1B in	-1.675

bioRxiv preprint doi: https://doi.org/10.1101/2024.02.13.580133; this version posted February 14, 2024. The copyright holder for this preprint (which was not certified by peer review) is the author/funder, who has granted bioRxiv a license to display the preprint in perpetuity. It is made available under aCC-BY 4.0 International license.

Table S4. Genes responsive to aripiprazole only. *C. albicans* was grown in RPMI-pH 7 at 35ºC for 6-hours with 5 μM of aripiprazole or 0.5% DMSO (vehicle). High throughput sequence analysis was performed, and significantly altered (log_2fold > 1 or < -1, adjusted to $p < 0.5$) gene expression (compared to vehicle controls) determined.

bioRxiv preprint doi: https://doi.org/10.1101/2024.02.13.580133; this version posted February 14, 2024. The copyright holder for this preprint (which was not certified by peer review) is the author/funder, who has granted bioRxiv a license to display the preprint in perpetuity. It is made available under aCC-BY 4.0 International license.

Gene name	Gene description	Log_2 of Average Fold Change
FRE30	Protein with similarity to ferric reductases; downregulated in response to amphotericin B, estradiol, or ciclopirox olamine, and upregulated by interaction with macrophage;	1.033
CTN3	Peroxisomal carnitine acetyl transferase; no obvious metabolic, hyphal, virulence defects in Ura+ strain; induced by macrophage engulfment, hyphal growth, starvation, nonfermentable carbon sources	1.021
FRE7	Copper-regulated cupric reductase; repressed by ciclopirox olamine or 17 -beta-estradiol; induced by alkaline conditions or interaction with macrophage	1.011
PHO100	Putative inducible acid phosphatase; DTT -extractable and observed in culture supernatant in low-phosphate conditions; slight effect on murine virulence	-1.52
GIT1	Glycerophosphoinositol permease; involved in utilization of glycerophosphoinositol as a phosphate source	-1.697
orf19.1123	Dubious open reading frame	-1.753
orf19.5262	Protein of unknown function	-3.505

Table S5. Genes responsive to etofenamate only. *C. albicans* was grown in RPMI-pH 7 at 35ºC for 6-hours with 5 µM of etofenamate or 0.5% DMSO (vehicle). High throughput sequence analysis was performed, and significantly altered (log_2fold > 1 or < -1, adjusted to $p < 0.5$) gene expression (compared to vehicle controls) determined.

bioRxiv preprint doi: https://doi.org/10.1101/2024.02.13.580133; this version posted February 14, 2024. The copyright holder for this preprint (which was not certified by peer review) is the author/funder, who has granted bioRxiv a license to display the preprint in perpetuity. It is made available under aCC-BY 4.0 International license.

Gene name	Gene description	Log_2 of Average Fold Change
snR57	C/D box small nucleolar RNA (snoRNA)	1.220
snR70	C/D box small nucleolar RNA (snoRNA)	-1.235
snR47	C/D box small nucleolar RNA (snoRNA)	-1.555
snR33a	H/ACA box small nucleolar RNA (snoRNA)	-1.562
orf19.6899	Putative oxidoreductase; mutation confers hypersensitivity to toxic ergosterol analog	-1.583
BTA1	Protein of unknown function	-1.598
GAP2	General broad specificity amino acid permease; ketoconazole, flucytosine repressed	-1.848
orf19.3378	Protein of unknown function; regulated by *TSA1*	-2.081
tG(GCC)6	RNA-Gly, predicted by tRNAscan -SE; GCC anticodon	-2.089
RAS2	Protein similar to *S. cerevisiae* RAS2;	-2.174
orf19.6688	Protein of unknown function; expression decreases by benomyl treatment or in an azole-resistant strain overexpressing MDR1	-2.389

Table S6. Genes responsive to mometasone only. *C. albicans* was grown in RPMI-pH 7 at 35ºC for 6-hours with 5 µM of mometasone or 0.5% DMSO (vehicle). High throughput sequence analysis was performed, and significantly altered (log_2fold > 1 or < -1, adjusted to $p < 0.5$) gene expression (compared to vehicle controls) determined.

bioRxiv preprint doi: https://doi.org/10.1101/2024.02.13.580133; this version posted February 14, 2024. The copyright holder for this preprint (which was not certified by peer review) is the author/funder, who has granted bioRxiv a license to display the preprint in perpetuity. It is made available under aCC-BY 4.0 International license.

Strain	Genotype	Source
SC5314	*C. albicans* reference strain	1
TACA12H10-pLUX-1	C. albicans his1Δ/Δ arg4Δ/Δ tac1Δ::ARG4/tac1Δ::HIS1 ura3Δ/Δ:URA3	This study
TACA12H10-pLUX-TAC1-1-1	C. albicans his1Δ/Δ arg4Δ/Δ tac1Δ::ARG4/tac1Δ::HIS1 ura3Δ/TAC1Δ:URA3	This study
UPC2M4A	*C. albicans upc2-1*Δ::FRT/ *upc2-2*Δ::FRT	2
UPC2M2A	*C. albicans upc2-1*Δ::FRT/ *UPC2*	2
CAI4+pKE4-NRG1	*ura3*Δ/Δ:*URA3*-Pr*TEF1-NRG1*	unpublished

Table S7. Strains used in this study.

Primer	Sequence
TAC1DISF	AATAAATTCAGATTCCCTTTCAGCCAAGAAAAAACTCCAAGAAAAGAAATAGAGCCTTT CTCCTTCTCTCTGTGGAATTGTGAGCGGATA
TAC1DISR	TGAAACAATAAATATTTACAAAGATATACATTATACATCGCTTTCACCAATTACAACTCT TTTTTAACCCGTTTTCCCAGTCACGACGTT
TAC1AMPF	TCAGGTACCGAGATGATGCAAAATCACACG
TAC1AMPR	TCAGAGCTCGGGGTGAGTATTGCTGTTTCCC

Table S8. Oligonucleotides used in this study.

References:

1. **Odds FC, Brown AJ, Gow NA.** Candida albicans genome sequence: a platform for genomics in the absence of genetics. Genome Biol. 2004;5(7):230. doi: 10.1186/gb-2004-5-7-230. Epub 2004 Jun 11. PMID: 15239821; PMCID: PMC463275
2. **Dunkel N, Liu TT, Barker KS, Homayouni R, Morschhäuser J, Rogers PD.** A gain-of-function mutation in the transcription factor Upc2p causes upregulation of ergosterol biosynthesis genes and increased fluconazole resistance in a clinical Candida albicans isolate. Eukaryot Cell. 2008 Jul;7(7):1180-90. doi: 10.1128/EC.00103-08. Epub 2008 May 16. PMID: 18487346; PMCID: PMC2446669.
